# Demystifying Experimental Parameters in Optical Photothermal Imaging in Aqueous Media

**DOI:** 10.1021/acsomega.6c10332

**Published:** 2026-09-25

**Authors:** Nikolaus Hondl, Nelson G. C. Astrath, Elisabeth Holub, Bernhard Lendl, Georg Ramer

**Affiliations:** † Institute of Chemical Technologies and Analytics, 27259 TU Wien, Vienna 1060, Austria; ‡ Department of Physics, Universidade Estadual de Maringá, Maringá, Paraná 87020-900, Brazil; § Christian Doppler Laboratory for Advanced Mid-Infrared Laser Spectroscopy in (Bio-)Process Analytics, TU Wien, 1060 Wien, Austria

## Abstract

Optical photothermal infrared (O-PTIR) spectroscopy is an indirect mid-infrared spectral imaging technique that enables label-free chemical analysis with submicrometer resolution. The technique is often used for analyzing cells and tissues and is discussed as capable of measuring in aqueous media, yet a quantitative understanding of its signal formation in liquids remains incomplete. Here, we present a systematic experimental study of transmission-mode O-PTIR supported by detailed simulations on the underlying signal generating process, focusing on O-PTIR applied to a water filled cuvette comprising two CaF_2_ windows with a path length of 600 μm, by finite element analysis. We investigate the influence of moving the focal spot of the excitation mid-IR beam from the window-liquid interface into the liquid and link excitation parameters to thermal dynamics and observed readout by the probe beam. We show that the signal amplitude is governed by the pulse duration, which controls the deposited thermal energy, while the modulation frequency determines the thermal diffusion length and thus the spatial extent of the response. Depth-resolved and probe-position-dependent measurements reveal a nonlocal photothermal response dominated by refractive index gradients associated with the photothermal lens effect involving both the liquid and the windows. Time-resolved analysis further separates the temperature field into a large steady-state (DC) component and a smaller oscillatory (AC) contribution, with the latter increasing at lower modulation frequencies and both decreasing with shorter pulse durations. These results establish a unified physical picture of O-PTIR signal generation and provide a basis for practical guidelines for optimizing sensitivity, spatial resolution, and quantitative performance in liquid-phase O-PTIR measurements.

## Graphical abstract

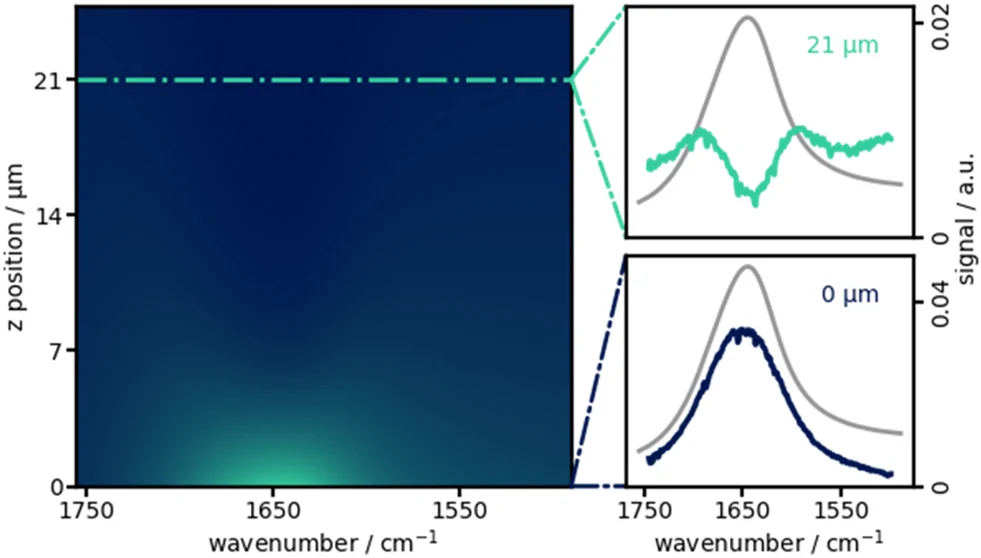

## 1 Introduction

Optical photothermal infrared (O-PTIR) spectroscopy is a nondestructive, label-free technique based on a pump–probe approach to detect molecular vibrations arising from light-matter interactions. A tunable mid-infrared (mid-IR) quantum cascade laser (QCL) serves as the excitation source, providing high brightness, selective vibrational contrast, and high sensitivity. Absorption of IR radiation induces localized heating in the sample, leading to a change in the refractive index.^1–4^

This photothermal response is probed by a tightly focused visible laser beam. The induced refractive index changes and, in some cases, surface displacement, modulate the probe beam via focusing or defocusing, which is detected as the photothermal signal. Because the probe operates in the visible range,^5–8^ O-PTIR enables lateral spatial resolution beyond the diffraction limit of conventional IR spectroscopy. In typical pump–probe configurations,^9–11^ spatially mismatched excitation and probe beams are used to enhance detection sensitivity.

O-PTIR has already been used in numerous applications for imaging of solid samples, ranging from polymers^12,13^ to nanoparticles^14–17^ and various cell types.^18–20^ These studies demonstrate the broad applicability of O-PTIR for analysis of dried samples. As cells are typically studied in water-based media, it is essential to understand how the O-PTIR signal behaves in this environment. Initial studies^21,22^ have shown the feasibility of O-PTIR in aqueous media. However, these studies do not discuss the influence of water absorption and experimental conditions on the O-PTIR signal.

In this study we leverage a custom-built O-PTIR setup based on a confocal coaxial design and counter propagation of mid-IR pump and visible readout beams via reflective optics and optimized for lab-on-a-chip applications.^23,24^ The system offers precise control over key experimental parameters, including repetition rate, pulse duration, adjustment of the probe beam focus and axial (z) position of the sample. Measurements were performed using a sample consisting of a 2 mm CaF_2_ window, a 600 μm thick aqueous layer, and a second 2 mm CaF_2_ cover layer. The confocal design enables measurements in water and allows controlled axial scanning to probe different offsets within the sample. Using this platform, we systematically investigate how focusing conditions and excitation parameters affect the O-PTIR absorption signal in aqueous media. Our results demonstrate that accurate focusing, combined with repetition rates significantly higher than those typically reported in the literature, is essential for reliable spectroscopy.

## 2 Experimental Method

The O-PTIR measurements were performed using a pump–probe configuration in a confocal transmission geometry, as illustrated in Figure 1. The pump beam was provided by an external-cavity quantum cascade laser (EC-QCL, MIRcat-QT-z, Daylight Solutions) tunable from 1480 cm^–1^ to 1750 cm^–1^ operated in pulsed mode, with a spectral resolution of 1 cm^–1^ and a scan rate of 100 cm^–1^ per second. The pulse width and repetition rate were set to several test values ranging from 10 to 500 kHz at 500 ns, respectively. The EC-QCL laser wavelength was swept continuously across the selected spectral range from lower to higher wavenumbers while the lock-in amplifier recorded the demodulated response as a function of time.

**Figure 1:**
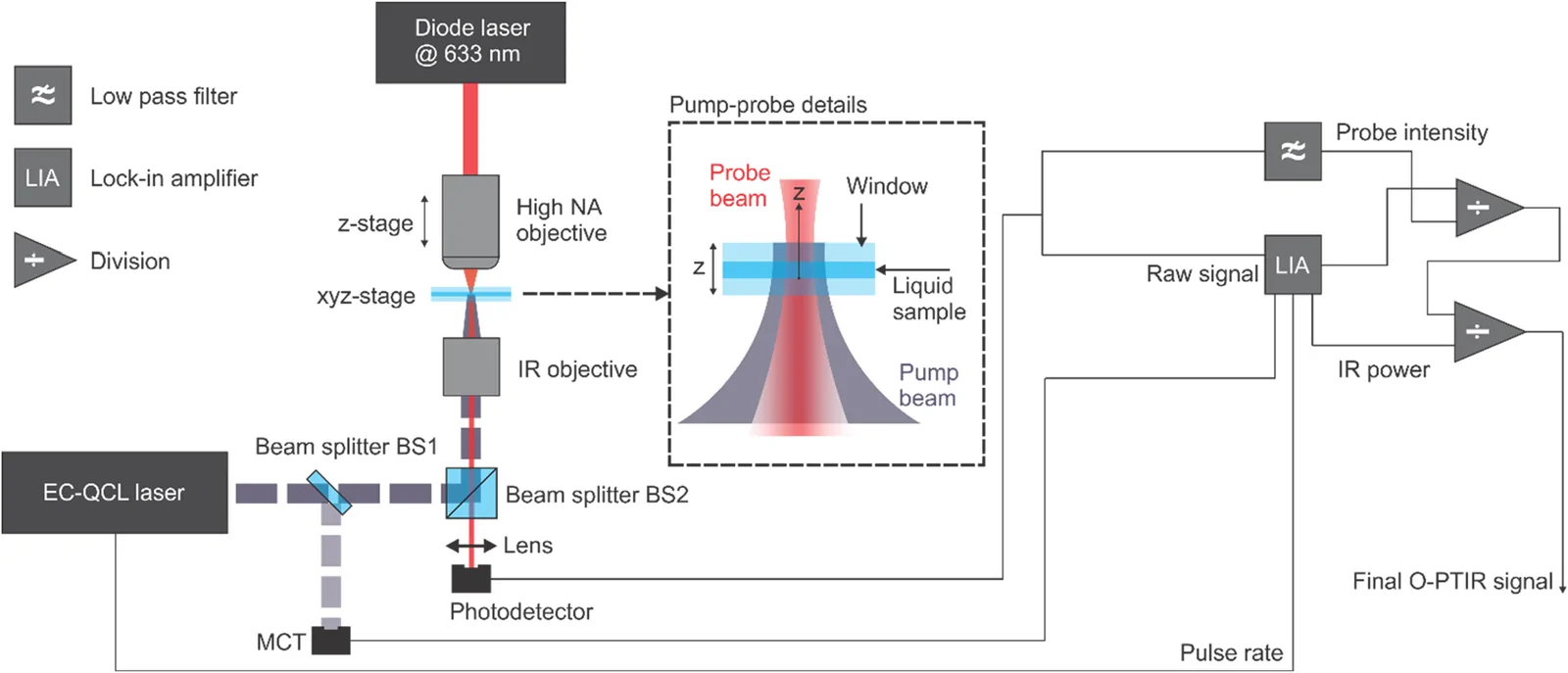
Schematic diagram of the O-PTIR experimental setup combining a 633 nm diode probe laser and a tunable EC-QCL pump beam. The beams are overlapped at the sample by two objectives, and the resulting photothermal signal is detected and processed using lock-in amplification to obtain the final O-PTIR signal.

Because the modulation frequencies employed here are in the kilohertz regime, the temporal spacing between excitation pulses is much shorter than the characteristic thermal relaxation time of the water/CaF_2_ assembly. Consequently, the sample does not fully cool between individual pulses nor between adjacent spectral points. Instead, repeated excitation leads to the buildup of a quasi-steady temperature field (DC component) on which a smaller oscillatory temperature modulation (AC component) is superimposed. The temperature field can therefore be written as *T*(**r**,t) = *T*_0_(**r**) + Δ*T*_DC_(**r**,**t**) + Δ*T*_AC_(**r**,t), where *T*_0_ is the ambient temperature, Δ*T*_DC_ is the nonoscillatory temperature increase that evolves monotonically toward a steady state, and Δ*T*_AC_ is the periodically modulated component at the laser repetition frequency.

The lock-in amplifier detects only the oscillatory part of the signal, i.e., the component oscillating at the modulation frequency. However, the DC temperature rise strongly influences the optical and thermal boundary conditions by establishing a persistent refractive index gradient and altering the baseline temperatures of the liquid and the windows.

The probe laser beam was a 633 nm diode laser (LBX-633, Oxxius) operated in cw mode. This instrument can be used for either single-point spectra, like in this study, or single-wavenumber images.^23^ The visible beam was focused on the sample by a 0.5 NA visible light objective (Olympus LMPLFLN, 50×, WD = 10.6 mm) with a probe power of approximately 0.5 mW at the sample. A manual micrometer screw and a sample position stage are used to adjust the probe beam focus. The mid-IR pump laser was focused on the back of the sample (ow2) using a reflective objective (Thorlabs LMM40X-PO, 40x, WD = 7.8 mm) with NA = 0.5, while a portion of the beam was reflected (BS1) onto a thermoelectrically MCT detector (PCI-2TE-12/MPAC-F-100, VIGO Systems S.A.) for power correction. The mid-IR pump beam remained fixed throughout the measurements. The axial overlap between the pump and probe beams was adjusted by translating the visible probe-beam focus with the movable probe objective. The sample stage allowed the sample to be moved in three dimensions independently of the pump and probe beam foci. Thus, depending on the experiment, either the probe focus or the sample position was varied, while the pump focus remained unchanged. The reflective objective was also used to collimate the probe beam after transmission through the sample. The transmitted visible light passed through the beam splitter (BS2) and was then focused onto a photodiode, where the photothermal signal was detected by a custom preamplifier (AMP) and then filtered by a lock-in amplifier (LIA, MFLI 500 kHz, Zurich Instruments). The LIA was used to demodulate the signal at the laser repetition rate with a fixed time constant of 5 ms. The excitation frequencies studied were in the kilohertz range and thus much higher than those applied in previous studies of mid-IR photothermal spectroscopy of liquids. When operating at excitation frequencies of a few hundred Hertz and below complete heating and cooling cycles had been observed and maximum signal-to-noise ratio obtained at excitation frequencies of only a few tens of Hertz.^25,26^ The infrared beam path was flushed with dry air to remove atmospheric water vapor. The O-PTIR spectra presented in this work were analyzed without baseline correction or spectral smoothing to preserve the measured spectral line shape and avoid potential processing-induced distortions.

## 3 Theory

### 3.1 Temperature Change

The sample and optical windows have finite dimensions and are homogeneous. The temperature change, ***T***_***i***_(***r***, ***z***, ***t***), where ***i*** stands for the material, in the liquid sample (***i*** = ***f***) surrounded by the CaF_2_ optical windows. Here, ***i*** = ***ow*** 1 denotes the optical window on the probe-beam side, corresponding to the upper side in Figure 1, and ***i*** = ***ow*** 2 denotes the opposite optical window on the excitation-beam side. The temperature field resulting from optical absorption is obtained by solving the heat-diffusion equations^27^

1 $$\rho_{i}c_{\mathrm{pi}}\frac{\partial T_{i}}{\partial t}-k_{i}\nabla^{2}T_{i}=Q_{i}\alpha_{i}e^{-2r^{2}/\omega_{e}^{2}}e^{-\alpha_{i}\left(z-z_{i}\right)}$$

***ρ_i_*** is the mass density, ***c***_***pi***_ is the specific heat, ***k***_***i***_ is the thermal conductivity, **α**_***i***_ is the optical absorption coefficient at the excitation wavenumber, ***t*** is time and **ω**_***e***_ = 10 **μm** is the radius of the Gaussian excitation beam at the sample window interface, modeled here as a Gaussian profile heat source. Because most of the excitation energy is absorbed within the first few micrometers of the water layer, the change in beam radius over the relevant absorption depth is negligible compared with the beam waist, and **ω**_***e***_(***z***) was therefore treated as constant. Since the problem is radially symmetric, the temperature and displacement depend only on the normal *z*- and radial *r*-coordinates. ***z***_***i***_ is the position of the first interface of each material. Assuming the origin of the coordinate system is at the first optical window/liquid interface, the positions become ***z***_***ow*** 1_ = – ***L***_1_ and ***z***_***ow*** 2_ = ***L***_2_. ***L***_1_ and ***L***_2_ are the thicknesses of the first and second optical windows, respectively. ***L*** is the thickness of the liquid layer enclosed by the two optical windows. The amplitude of the Gaussian heat source is ***Q***_***f***_ = 2 ***P***_0_/**πω**_***e***_^2^. The optical absorption in the optical windows surrounding the liquid can be neglected, i.e., **α**_***ow*** 1_ = **α**_***ow*** 2_ = 0. ***P***_0_ = 100 **mW** is the peak power of the excitation beam used in the simulations.

### 3.2 Finite Element Analysis

Finite element analysis (FEA) software was used to compute numerical solutions to the heat diffusion equation, incorporating realistic boundary conditions derived from the experimental geometry. The model was created in 2D axisymmetric form using COMSOL Multiphysics 5.6. The “Heat Transfer in Solids” module was employed to determine the temperature distribution within the liquid sample and surrounding CaF_2_ optical windows. The values of the thermal, mechanical, and optical properties used in the FEA simulations are detailed in Flizikowski et al.^28^ The sample geometry was modeled corresponding to the experimental conditions, with optical windows measuring 2 mm in thickness and 12.7 mm in radius, along with a water layer of 600 μm thickness. The heat diffusion eq (eq 1) is solved across all domains. The model features four types of boundaries: one along the axis of symmetry at *r* = 0, which aligns with the center of the excitation and probe laser beams, and the exterior boundaries are set as thermally insulated.

Thermal convection was neglected in the simulations. Given the modulation frequencies used in this work (30 Hz–500 kHz), the characteristic thermal diffusion times (2 μs–0.7 ms) and diffusion lengths (0.3–40 μm) are far too short for buoyancy-driven flow to contribute measurably to heat transport within a single excitation cycle (single pulse or pulse block). The corresponding Rayleigh number remains well below the critical threshold for the onset of natural convection at these length scales. Heat transport is therefore diffusion-dominated on the time scale relevant for the O-PTIR signal, justifying the use of a pure conduction (“Heat Transfer in Solids”) model.

### 3.3 Propagation of Electromagnetic Radiation of the Excitation Beam

To analyze how electromagnetic radiation propagates through the aqueous medium, we employ the complex refractive index and the Transfer Matrix Method (TMM).^29,30^ The complex refractive index spectra (*n*(*ν̃*) = *n*(*ν̃*) + *ik*(*ν̃*)) of water and CaF_2_ were obtained from literature.^31,32^

The attenuation of light within the medium is quantified using the linear absorption coefficient. The linear Napierian absorption coefficient, α_*e*_, is calculated from the absorption *k* and the wavenumber *ν̃* as follows

2 $$\alpha_{e}\left(\mathrm{\nu ̃}\right)=4\pi \mathrm{\nu ̃}k\left(\mathrm{\nu ̃}\right)$$

α_e_(*ν̃*) provides a measure of the energy loss per distance as the wavefront propagates through the water layer. The local absorbed power density within the multilayer stack, specifically at the interface between the CaF_2_ substrate and the water layer, is calculated using a position-resolved TMM. For a given depth *z* and wavenumber *ν̃*, the local absorbed power *Q*(*z*,*ν̃*) is determined by

3 $$Q\left(z,\mathrm{\nu ̃}\right)=\frac{1}{2}c\epsilon_{0}\alpha_{e}\left(\mathrm{\nu ̃}\right)n\left(\mathrm{\nu ̃}\right){\left|E\left(z,\mathrm{\nu ̃}\right)\right|}^{2}$$

where *c* is the speed of light, ϵ_0_ is the permittivity, and *|E*(*z*,*ν̃*)*|* ^2^ ≈ *|E*_*F*_ e^*ik*_*f*_ *z*^ + *E*_*B*_ **e ^–*ik*_*f*_ *z*^ *|* ^2^ is the local electric field. *E*_*F*_ is the forward electric field and *E*_*B*_ the backward field. *k*_*f*_ = 2π*n*/λ_vac_ is the wavevector and λ_vac_ is the vacuum wavelength. This approach accounts for coherent effects and multiple reflections at the boundaries of the CaF_2_ substrate and water layer. In the case of a strongly absorbing, thick sample (3) simplifies to

4 $$Q\left(z,\mathrm{\nu ̃}\right)=\frac{1}{2}c\epsilon_{0}\alpha_{e}\left(\mathrm{\nu ̃}\right)n\left(\mathrm{\nu ̃}\right){\left|E_{F}\left(0,\mathrm{\nu ̃}\right)\right|}^{2}e^{-\alpha_{e}\left(\mathrm{\nu ̃}\right)z}$$

### 3.4 Effects of Absorption Induced Local Heating at Kilohertz Frequencies (AC and DC Signal)

The modulation frequency determines the time available for heat to diffuse away from the region of optical absorption during each excitation cycle and together with the thermal diffusion coefficient determines the length it can diffuse. This behavior can be described in terms of the thermal diffusion length, which increases as the modulation frequency decreases. At lower frequencies, the longer time between successive pulses allows heat to propagate further into the surrounding medium, resulting in a larger thermally perturbed area. Consequently, the induced refractive index gradient spans a large spatial region, effectively strengthening the photothermal lens effect. In contrast, at higher modulation frequencies, the time available for heat diffusion within each cycle is significantly reduced. As a result, the thermal energy remains confined to a smaller volume near the excitation region, producing a weaker temperature gradient. The corresponding refractive index change is therefore limited in both magnitude and spatial extent, reducing the efficiency of photothermal lens formation and resulting in a lower signal. Under these conditions, the system approaches a regime in which thermal confinement dominates, and the photothermal response becomes increasingly restricted to the vicinity of the absorption site.

In this scenario, the characteristic thermal diffusion time can be estimated from τ_th_ ≈ *d* ^2^/*D*_*i*_, where *d* is the characteristic diffusion distance and *D*_*i*_ is the thermal diffusivity of material *i*, defined as $D_{i}=\frac{k_{i}}{\rho_{i}c_{p,i}}$ . Typical thermal diffusivities are approximately *D*_water_ ≈ 1.4 × 10^–7^ m^2^/s and *D*_CaF_2__ ≈ 3.7 × 10^–6^ m^2^/s. Thus, heat propagates much faster inside the CaF_2_ windows than inside water. For a thermal diffusion distance of *d* ∼ 10 μm, representative of the excitation region, the thermal equilibration time in water is on the order of τ_water_ ≈ 0.7 ms while in CaF_2_ it is τ_CaF_2__ ∼ 0.03 ms. These values are crucial when compared with the modulation periods of, for instance, *T*_30Hz_ = 33 ms and *T*_500kHz_ = 2 μs.

At 30 Hz, the excitation period is much longer than the thermal diffusion time. The system therefore undergoes nearly complete heating/cooling cycles during each modulation period, minimizing thermal accumulation. At 500 kHz, however, the modulation period is several hundred times shorter than the characteristic diffusion time. Under these conditions, heat cannot dissipate between pulses, resulting in substantial steady-state (DC) heat accumulation.

The thermal diffusion length, μth=Dπf , is approximately ∼40 μm for water at 30 Hz, but only ∼0.3 μm at 500 kHz. Thus, low-frequency excitation generates large spatially extended thermal gradients, whereas high-frequency excitation produces strong thermal confinement near the absorption region.

## 4 Results and Discussion

Supporting Figure S1 shows the simulated temperature response at the CaF_2_/water interface for different excitation frequencies and pulse durations. The temperature field consists of a monotonically increasing DC component and a smaller oscillatory AC component at the modulation frequency. As the modulation frequency decreases, the amplitude of these oscillations grows, because the system has more time to allow heat to spread and accumulate between pulses, leading to stronger thermal gradients and a more pronounced photothermal lens effect. Shorter pulse durations reduce both DC and AC temperature changes because less energy is deposited per pulse, resulting in lower overall heating and a weaker photothermal response (Figure S1b). Together, these results demonstrate that modulation frequency and pulse duration jointly control how heat accumulates and dissipates. Their interplay ultimately determines the magnitude of the temperature gradients and, therefore, the strength and behavior of the O-PTIR photothermal response.

Figure S2 compares the normalized O-PTIR transient signals measured at the CaF_2_/water interface and at focal positions inside the water layer with the corresponding simulated transients at an excitation wavenumber of 1660 cm^–1^. No analytical model was fitted to the experimental data. Instead, the early time response was characterized by *t*_90_, defined as the time required for the normalized transient to reach 90% of its value within the investigated time window. The experimental *t*_90_ values were (138 ± 39) μs, (133 ± 45) μs, and (122 ± 33) μs at the interface and at focal offsets of 14 and 28 μm, respectively, where the uncertainties represent one standard deviation across 100 measurements. The corresponding simulated values were 165, 163, and 156 μs. Thus, both the experimental and simulated transients exhibit an initial step-like increase on a comparable time scale of approximately 120–165 μs, indicating that the simulation captures the characteristic early time response of the system. The differences between the mean experimental *t*_90_ values are small compared with their variability, consistent with only a weak dependence of the transient dynamics on focal position.

Figure 2 presents the results of the experimental depth profile of the O-PTIR spectra at a fixed frequency of 500 kHz and a pulse width of 500 ns compared to calculated, depth resolved absorbed energy fractions per voxel. The stated *z*-values in μm of the displayed depth resolved O-PTIR spectra correspond to the distance of the focal point of the visible probe beam with respect to the window (CaF_2_)—sample (water) interface. When the excitation laser beam is focused on the CaF_2_/water interface, the recorded O-PTIR spectrum strongly depends on the absorption coefficient of water and thus resembles the water absorption spectrum. As the probed volume is moved from the interface into the water layer (by moving the sample downward) a dip begins to appear in the center of the water band. This behavior is due to the high absorption coefficient (around 0.28 μm^–1^) at the center of the absorption band (1645 cm^–1^).

**Figure 2:**
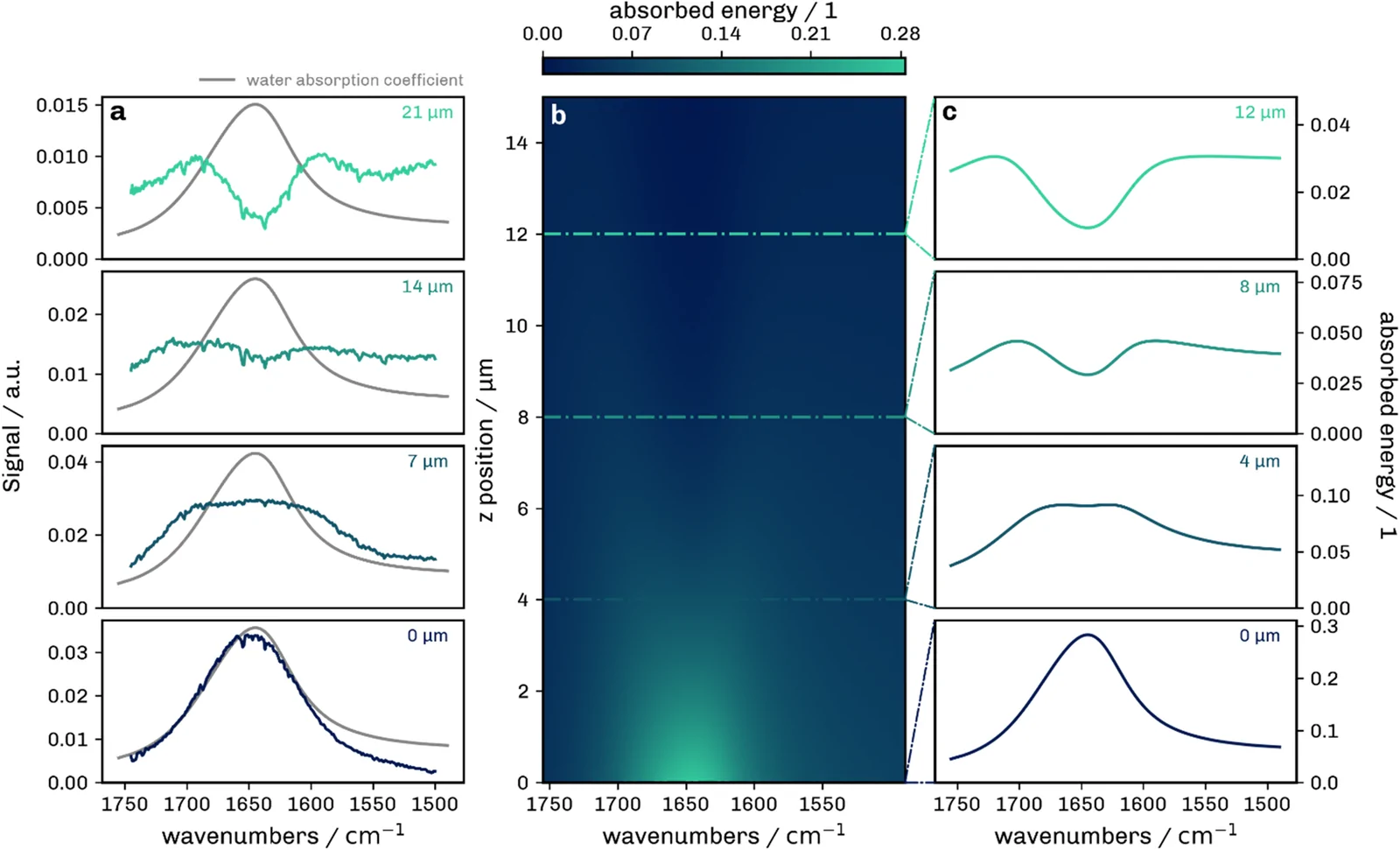
(a) Depth-dependent O-PTIR spectra recorded at a modulation frequency of 500 kHz and a pulse width of 500 ns. The indicated depths correspond to the position of the visible probe-beam focus relative to the CaF_2_/water interface. The spectra are shown without baseline correction or spectral smoothing. The light-gray curve represents the absorption coefficient of water. (b) Numerically calculated local absorbed excitation power according to eq 3. (c) Depth-resolved cross sections of the calculated absorbed-power distribution shown in (b).

This means that at the center of the water bending vibration, 3.6 μm from the interface, the remaining light intensity and therefore the local absorbed power at the center has dropped to 1/e of the local absorbed power at the interface (and to 1/10 after 8.2 μm). While at the interface the local absorbed power is lower at the sides than at the center, the intensity at the flanks of the water absorption band drops more slowly with *z*. Therefore, eventually, as z increases, the local absorbed power at the center of the band is lower than that on the band’s flanks due to the more rapid light intensity decay at the center.

Note that the calculated distribution of absorbed power (Figure 2c) at certain distances from the window-water interface resembles the recorded experimental spectra when the sample is moved and the focus of the visible probe beam is located at the same depths. Due to the distribution of the intensity field along the *z*-axis the shape of the recorded O-PTIR spectra also changes as a function of different depths. Considering the time scale of vibrational–translational relaxation in liquids, heat diffusion and the applied modulation frequencies in the kilohertz range, the distribution of the intensity field reflects a quasi-synchronously generated refractive index field. In particular, the fact that the changes of the O-PTIR signal with depth follow the change observed in the calculated profiles confirms that by means of a highly focused probe beam, as present in this setup, O-PTIR signals along the *z*-axis can be recorded, which are governed by the spatial distribution of absorbed energy. It is interesting to note that the distribution of absorbed energy as a function of distance from the surface will deviate from the absorption spectrum of the sample, the more the power has already been reduced by absorption within the sample. This is the case for liquid water, which is clearly visible in the center of the water bending vibration at increasing distances from the window-sample interface. The observed changes in spectral shape with increasing depth can therefore be understood as a consequence of the depth-dependent intensity field.

Near the interface, the excitation is strong and sharply localized, producing well-defined spectral features in-line with the absorbance spectrum of the sample. At greater depths, the reduced and spatially broadened intensity results in weaker, slightly altered spectral profiles. The signal is not generated at the geometric focus of the visible probe beam.^23^ Instead, the probe beam integrates phase distortions accumulated along its entire propagation path through the thermally perturbed refractive index field. The important point is that the detected quantity is not the local temperature itself, but rather the wavefront distortion imposed on the probe beam by the refractive index distribution *n*(*r*,*z*). The measured signal therefore reflects the integrated effect of the refractive index field along the propagation direction *z*, rather than a local interaction at the beam waist. The optical phase accumulated by the probe beam is given by

5 $$\Delta \phi =\frac{2\pi }{\lambda }\int \Delta n\left(r,z\right)\mathrm{dz}$$

where Δ*n*(*r*,*z*) is the temperature-induced refractive index change and λ is the probe wavelength. Since the refractive index perturbation extends over tens of micrometers due to thermal diffusion, the probe beam experiences phase modulation over a substantial propagation distance. This agreement between experiment and model demonstrates that the O-PTIR signal can be interpreted as the combination of the absorption coefficient with the spatial distribution of the squared excitation field, as described by eq 3.

Importantly, these results also emphasize that the O-PTIR signal is not solely determined by local absorption at a specific depth, but rather by the integrated effect of the intensity field over a finite volume defined by optical propagation and thermal diffusion. The depth-dependent evolution of the spectra therefore reflects both the attenuation of the excitation beam and the resulting spatial distribution of the photothermal source term. Overall, Figure 2 provides strong experimental validation of the “propagation of electromagnetic radiation” model and establishes a clear connection between electromagnetic energy deposition, thermal generation, and the resulting photothermal lens signal detected in O-PTIR measurements.

The principal depth-dependent change in the measured spectra is the development of a dip near the center of the water bending band, which is consistent with the calculated redistribution of the absorbed excitation energy. A smaller asymmetry between the low- and high-wavenumber sides of the experimental spectra is also observed. However, this asymmetry cannot be explained solely by the wavelength dependence of the water absorption coefficient, since wavenumbers with similar absorption coefficients do not always produce identical O-PTIR signal amplitudes. This indicates that the measured spectral line shape is influenced not only by the absorption coefficient but also by the spatial distribution of the excitation field and the resulting thermally induced refractive-index perturbation. Because the probe beam accumulates phase distortions along its propagation path, differences in the three-dimensional heat-source and refractive-index distributions may lead to different signal amplitudes even at wavenumbers with similar absorption coefficients. Additional wavelength-dependent experimental contributions cannot be excluded.

Another source of this mismatch could be contribution from cumulative heating during spectral acquisition. Under kilohertz excitation, incomplete cooling between successive excitation pulses produces a slowly varying temperature component in addition to the modulated photothermal response. However, the transient measurements shown in Figure 2S indicate that the initial step-like response develops within approximately 150 μs in the studied volume. Contributions associated with spectral scan history are therefore expected to be secondary to the depth-dependent redistribution of the excitation energy.

Figure 3 presents the dependence of the O-PTIR signal on the modulation frequency, i.e., the pulse repetition rate of the pump beam, with the system focused on the CaF_2_/water interface and at a nominal axial offset of 7 μm within the water layer, while maintaining a constant pulse width of 500 ns. The dependence of the signal amplitudes on modulation frequency reflects the fundamental role of thermal diffusion in governing photothermal lens formation, although the dependence is not strictly monotonic.

**Figure 3:**
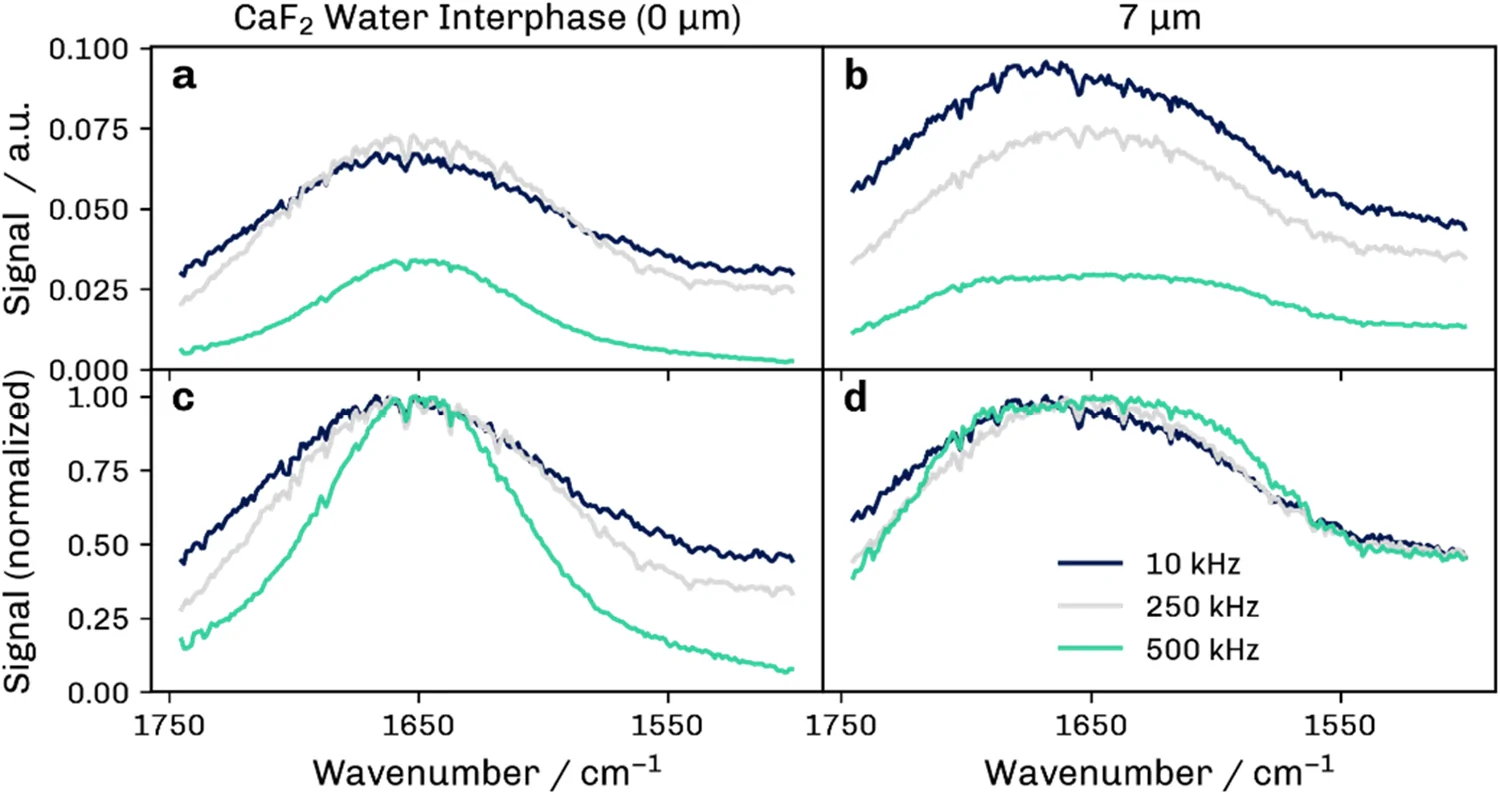
O-PTIR signal at the surface of CaF_2_/water (panels a and c) and at 7 μm (panels b and d) deep in the water for different modulated frequencies with a fixed pulse width of 500 ns. To better show the difference in shape of the signals they are also presented scaled to have a maximum value of 1 (panels c and d).

The modulation frequency determines the characteristic time available for heat to diffuse away from the region of optical absorption during each excitation cycle. This behavior can be described in terms of the thermal diffusion length, which increases as the modulation frequency decreases. At lower frequencies, the longer time between successive pulses allows heat to propagate further into the surrounding medium, resulting in a larger thermally perturbed area. Consequently, the induced refractive index gradient spans a large spatial region, affecting the photothermal lens. In contrast, at higher modulation frequencies, the time available for heat diffusion within each cycle is significantly reduced. As a result, the thermal energy remains confined to a smaller length of about 30 μm near the excitation region, thereby reducing the temperature gradient. The corresponding refractive index change is therefore limited both in magnitude and spatial extent, reducing the effective interaction volume. Under these conditions, the system approaches a regime in which thermal confinement dominates, and the photothermal response becomes increasingly restricted to the vicinity of the absorption site.

The comparison between the signals recorded at the interface and the nominal axial offset of 7 μm further highlights the interplay between optical absorption and thermal transport (see Figure 3). At the CaF_2_/water interface, where the absorbed energy is highest and proportional to the linear absorption coefficient of the sample due to minimal prior attenuation, the generated temperature rise is larger. At a nominal position of 7 μm, however, the excitation beam is partially attenuated by absorption, resulting in reduced heat generation. Nevertheless, at this depth the signal at lower modulation frequencies is still larger than at the interphase, demonstrating that it is not only the local absorption that defines the signal. Even though the laser modulation frequency affects the shape of the spectrum at this depth as well, none of the tested frequencies reproduces the water absorption spectrum. These results emphasize that the O-PTIR signal is critically governed by the balance between heat generation and heat dissipation. Lower modulation frequencies favor efficient heat accumulation and diffusion, leading to larger temperature gradients and stronger photothermal lensing, whereas higher frequencies restrict thermal transport and reduce the effective interaction volume. However, the resulting signal amplitude is not strictly monotonic because it also depends on the pump–probe geometry.

The spectral behavior across frequencies indicates that, while the amplitude of the signal is strongly frequency-dependent, the underlying absorption characteristics of the sample remain unchanged. This confirms that the modulation frequency primarily controls the efficiency and spatial extent of photothermal lens formation, rather than altering the chemical specificity of the measurement. Only when probing at the interface and using a 500 kHz repetition rate—a rate significantly higher than repetition rates typically used in O-PTIR^5,22,23,33^ —is the shape of the O-PTIR signal comparable to the water absorption coefficients across the studied spectral range.

The influence of the probe-beam position on the detected signal was also investigated. Prior to acquiring the data shown in Figure 4, the relative axial alignment of the pump and probe beams was optimized to obtain the maximum signal; this position was defined as 0 μm offset. The probe focus was then displaced axially relative to the fixed pump focus while the sample position remained unchanged. Figure 4, illustrates the dependence of the detected photothermal signal on the relative axial pump–probe offset within the absorbing water layer. This behavior is consistent with a previous study,^23^ which showed that the axial photothermal response is strongly dependent on the relative positions of the pump and probe foci and exhibits a non-Gaussian profile. Consequently, the signal is not expected to decrease monotonically as the axial offset increases.

**Figure 4:**
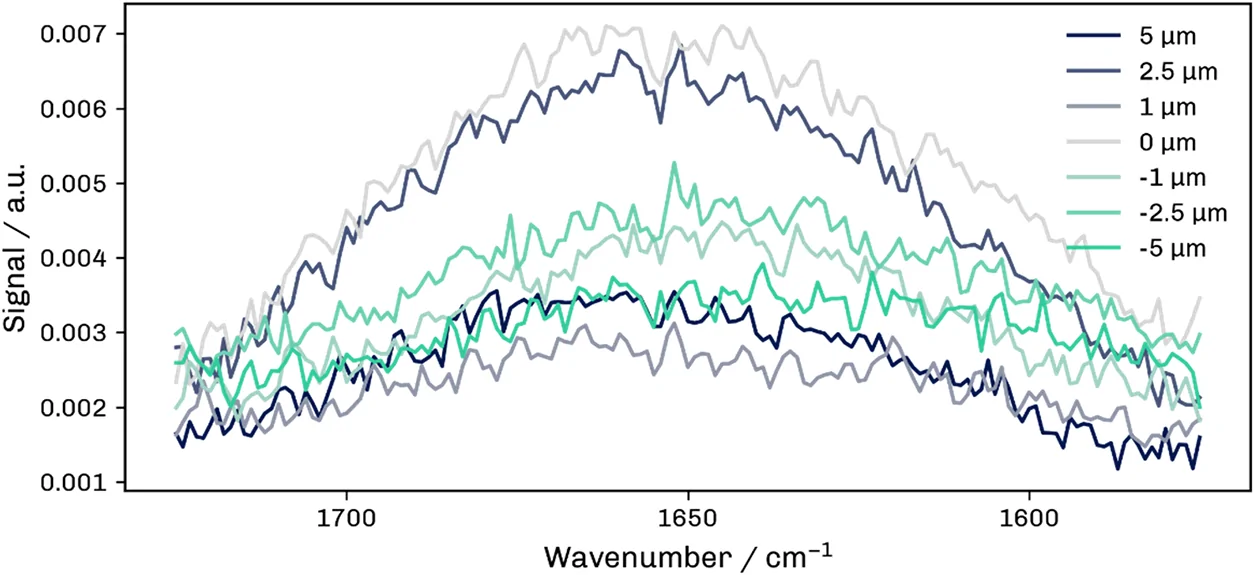
O-PTIR signal at 500 kHz repetition rate and 500 ns pulse width for different probe beam positions in the sample. The figure shows that the photothermal spectrum depends on the relative axial positions of the pump and probe foci.

## 5 Conclusions

We have established a quantitative framework for understanding photothermal signal formation in transmission-mode O-PTIR by linking excitation conditions to thermal transport and measured response. The signal amplitude is governed by deposited thermal energy, while thermal diffusion defines both the magnitude and spatial extent of the response, revealing the fundamentally nonlocal character of the technique.

Accurate spectroscopy in strongly absorbing, wavelength-dependent backgrounds is achieved only by probing the immediate vicinity of the interface. At greater offsets, attenuation of the excitation field distorts spectral features. Modulation frequency is the key control parameter: increasing frequency confines thermal diffusion and shrinks the effective probed volume, enabling accurate recovery of the water absorption band only at frequencies ≫ 100 kHz. Particularly, operation at 500 kHz, well beyond commonly used O-PTIR repetition rates (∼50–100 kHz) for imaging of solid samples and also of previous photothermal liquid spectroscopy (below 500 Hz), is required to access this regime.

The thermal response separates into a dominant steady-state (DC) component and a smaller oscillatory (AC) contribution. The AC component increases at lower frequencies due to extended diffusion times, enhancing photothermal lensing, whereas both components decrease with shorter pulse durations due to reduced energy deposition per cycle. In addition, signal detection depends critically on probe–pump overlap; maintaining a constant probe offset is therefore essential for reproducible measurements. A crucial practical implication is that O-PTIR in absorbing media induces substantial steady-state heating, with temperature rises sufficient to damage sensitive samples (up to 50° K in our simulations).

Taken together, these results identify modulation frequency and pulse duration as the primary levers controlling energy deposition and thermal transport and establish photothermal lensing as the dominant signal mechanism in liquids. More broadly, they redefine O-PTIR in aqueous environments as an interface-sensitive, thermally delocalized measurement, setting clear boundaries for quantitative spectroscopy while opening a pathway to controlled, high-resolution chemical imaging in strongly absorbing media. In short, this framework provides these practical guidelines for optimizing sensitivity, spatial resolution, and quantitative performance:

1. In spectral ranges with strong wavelength-dependent background absorption, correct spectra can only be acquired when probing the volume right at the interface.
2. Higher modulation frequencies shrink the probed volume. Only for modulation frequencies ≫ 100 kHz the water absorption band is recovered accurately.
3. The relative position of the probe beam focus affects the signal. As further study is required to quantify this effect, we recommend that the same offset should be used throughout a study.
4. O-PTIR in absorbing media can induce significant DC heating of the sample. Typical values are sufficient to destroy sensitive specimens.

Whereas extraction of analyte (more specifically solute) characteristic spectra within the sample appears not possible at this stage it might become possible in the future. For this to happen a complete simulation of all light matter interactions within the sample along the whole excitation beam path might open possibilities to extract the different and spatially distributed absorption coefficients from such measurements by means of advanced fitting routines.

## Supplementary Material



## Data Availability

The data underlying this study are openly available in Zenodo at DOI: 10.5281/zenodo.19693993.

## Notes

Simulated temperature response at the CaF_2_/water interface for different excitation frequencies and pulse durations (Figure S1); experimental and simulated O-PTIR transients at the CaF_2_/water interface and inside the water phase (Figure S2) (PDF)

## References

[1] Lee E. S., Lee J. Y. (2011). High Resolution
Cellular Imaging with Nonlinear Optical
Infrared Microscopy. Opt. Express.

[2] Li C., Zhang D., Slipchenko M. N., Cheng J.-X. (2017). Mid-Infrared Photothermal
Imaging of Active Pharmaceutical Ingredients at Submicrometer Spatial
Resolution. Anal. Chem..

[3] Kansiz M., Prater C., Dillon E., Lo M., Anderson J., Marcott C., Demissie A., Chen Y., Kunkel G. (2020). Optical Photothermal
Infrared Microspectroscopy with Simultaneous Raman – A New
Non-Contact Failure Analysis Technique for Identification of <
10 Μm Organic Contamination in the Hard Drive and Other Electronics
Industries. Microsc. Today.

[4] Xia Q., Yin J., Guo Z., Cheng J.-X. (2022). Mid-Infrared Photothermal Microscopy:
Principle, Instrumentation, and Applications. J. Phys. Chem. B.

[5] Prater C. B., Kansiz M., Cheng J.-X. (2024). A Tutorial
on Optical Photothermal
Infrared (O-PTIR) Microscopy. APL Photonics.

[6] Furstenberg, R. ; Kendziora, C. A. ; Papantonakis, M. R. ; Nguyen, V. ; McGill, R. A. In Chemical Imaging Using Infrared Photothermal Microspectroscopy, SPIE Proceedings; Druy, M. A. ; Crocombe, R. A. , Eds.; SPIE: Baltimore, Maryland, USA, 2012 837411 10.1117/12.919574.

[7] Mërtiri, A. ; Totachawattana, A. ; Liu, H. ; Hong, M. K. ; Gardner, T. ; Sander, M. Y. ; Erramilli, S. In Label Free Mid-IR Photothermal Imaging of Bird Brain With Quantum Cascade Laser, CLEO; OSA: San Jose, CA, 2014 AF1B.4 10.1364/CLEO_AT.2014.AF1B.4.

[8] Olson N. E., Xiao Y., Lei Z., Ault A. P. (2020). Simultaneous Optical
Photothermal Infrared (O-PTIR) and Raman Spectroscopy of Submicrometer
Atmospheric Particles. Anal. Chem..

[9] Marcano, O. A. ; Cabrera, M. H. ; Díaz, B. M. ; Wetter, N. U. ; Frejlich, J. In Comparison Between Mode-Matched and Mode-Mismatched Thermal Lens Methods for Absorption Measurements in Liquids, AIP Conference Proceedings; AIP: Campinas, São Paulo (Brazil), 2008; Vol. 992, pp 1189–1194 10.1063/1.2926816.

[10] Baesso M. L., Shen J., Snook R. D. (1994). Mode-Mismatched
Thermal Lens Determination
of Temperature Coefficient of Optical Path Length in Soda Lime Glass
at Different Wavelengths. J. Appl. Phys..

[11] Andrade A.
A., Catunda T., Bodnar I., Mura J., Baesso M. L. (2003). Thermal
Lens Determination of the Temperature Coefficient of Optical Path
Length in Optical Materials. Rev. Sci. Instrum..

[12] Böke J. S., Popp J., Krafft C. (2022). Optical Photothermal Infrared Spectroscopy
with Simultaneously Acquired Raman Spectroscopy for Two-Dimensional
Microplastic Identification. Sci. Rep..

[13] Gruber E. S., Karl V., Duswald K., Bhamidipalli M. S., Schlederer M., Limberger T., Kopatz V., Teleky B., Kenner L., Brandstetter M. (2025). Method for Label-Free & Non-Destructive
Detection of Microplastics in Human Formalin-Fixed Paraffin-Embedded
Tissue Sections. Sci. Rep..

[14] Boyer D., Tamarat P., Maali A., Lounis B., Orrit M. (2002). Photothermal
Imaging of Nanometer-Sized Metal Particles Among Scatterers. Science.

[15] Zahedian M., Koh E. S., Dragnea B. (2019). Photothermal
Microspectroscopy with
Bessel–Gauss Beams and Reflective Objectives. Appl. Opt..

[16] Selmke M., Braun M., Cichos F. (2012). Gaussian Beam Photothermal Single
Particle Microscopy. J. Opt. Soc. Am. A.

[17] Berciaud S., Cognet L., Blab G. A., Lounis B. (2004). Photothermal Heterodyne
Imaging of Individual Nonfluorescent Nanoclusters and Nanocrystals. Phys. Rev. Lett..

[18] Yurdakul C., Zong H., Bai Y., Cheng J.-X., Selim
Ünlü M. (2021). Bond-Selective Interferometric Scattering Microscopy. J. Phys. Appl. Phys..

[19] Zhang D., Li C., Zhang C., Slipchenko M. N., Eakins G., Cheng J.-X. (2016). Depth-Resolved
Mid-Infrared Photothermal Imaging of Living Cells and Organisms with
Submicrometer Spatial Resolution. Sci. Adv..

[20] Bai Y., Camargo C. M., Glasauer S. M. K., Gifford R., Tian X., Longhini A. P., Kosik K. S. (2024). Single-Cell
Mapping of Lipid Metabolites
Using an Infrared Probe in Human-Derived Model Systems. Nat. Commun..

[21] Shuster, S. O. ; Curtis, A. E. ; Davis, C. M. Optical Photothermal Infrared Imaging Using Metabolic Probes in Biological Systems Biophysics 2024; Vol. 97 10.1101/2024.09.19.613881. PMC12270000 40207400

[22] Spadea A., Denbigh J., Lawrence M. J., Kansiz M., Gardner P. (2021). Analysis of
Fixed and Live Single Cells Using Optical Photothermal Infrared with
Concomitant Raman Spectroscopy. Anal. Chem..

[23] Holub E., Hondl N., Wöhrer S., Lendl B., Ramer G. (2025). Not Just Better
Resolution: A Detailed Study of the Signal Distribution in Mid-Infrared
Optical Photothermal Imaging. Anal. Chem..

[24] Holub E., Hondl N., Lin K.-L., Parikainen M., Sahlgren C., Lendl B., Ramer G. (2026). Investigating Spectral
Biomarker Candidates for Migratory Potential in Cancer Cells Using
Micro-FTIR and O-PTIR Spectroscopy. ACS Meas.
Sci. Au.

[25] Ricchiuti G., Dabrowska A., Pinto D., Ramer G., Lendl B. (2022). Dual-Beam
Photothermal Spectroscopy Employing a Mach–Zehnder Interferometer
and an External Cavity Quantum Cascade Laser for Detection of Water
Traces in Organic Solvents. Anal. Chem..

[26] Kau-Wacht D., Astrath N. G. C., Lukasievicz G. V. B., Lindenbauer L., Dabrowska A., Wieland K., Lendl B. (2025). Laser-Based Mid-IR
Photothermal Spectroscopy of Liquids: A New Avenue for in-Line Sensing
in Process Analytical Technology. Anal. Bioanal.
Chem..

[27] Lukasievicz G. V. B., Herculano L. S., Sehn E., Belançon M. P., Bialkowski S. E., Capeloto O. A., Astrath N. G. C., Malacarne L. C. (2020). An Experimental
Investigation of Sample–Fluid Heat Coupling Effect in Thermal
Lens Technique. Appl. Spectrosc..

[28] Flizikowski G. A. S., Anghinoni B., Rohling J. H., Belançon M. P., Mendes R. S., Baesso M. L., Malacarne L. C., Požar T., Bialkowski S. E., Astrath N. G. C. (2020). Influence of
Edge Effects on Laser-Induced Surface Displacement of Opaque Materials
by Photothermal Interferometry. J. Appl. Phys..

[29] Byrnes, S. J. Multilayer Optical Calculations. 2020, arXiv:1603.02720. arXiv.org e-Print archive. https://arxiv.org/abs/1603.02720 10.48550/arXiv.1603.02720.

[30] Byrnes, S. J. Tmm 2024, https://pypi.org/project/tmm/. (accessed 2026–04–05).

[31] Hale G. M., Querry M. R. (1973). Optical Constants of Water in the 200-Nm to 200-Μm
Wavelength Region. Appl. Opt..

[32] Malitson I. H. (1963). A Redetermination
of Some Optical Properties of Calcium Fluoride. Appl. Opt..

[33] Al
Jedani S., Lima C., Smith C. I., Gunning P. J., Shaw R. J., Barrett S. D., Triantafyllou A., Risk J. M., Goodacre R., Weightman P. (2024). An Optical
Photothermal Infrared Investigation of Lymph Nodal Metastases of Oral
Squamous Cell Carcinoma. Sci. Rep..

