# Supplementary material for: Demystifying Experimental Parameters in Optical Photothermal Imaging in Aqueous Media
Source: ACS Omega. 2026 Sep 25;11(39):60485–93. doi: 10.1021/acsomega.6c10332 (PMC13647896; doi:10.1021/acsomega.6c10332)
Supplement: Supplementary file 1 [file ao6c10332_si_001.pdf]

Supporting Information for “Demystifying Experimental Parameters in Optical Photothermal Imaging in Aqueous Media”

Nikolaus Hondl[1,3], Nelson G. C. Astrath[1,2], Elisabeth Holub[1,3], Bernhard Lendl[1], and Georg Ramer[1,3*]

[1] Institute of Chemical Technologies and Analytics, Technische Universität Wien, Vienna, 1060, Austria.
[2] Department of Physics, Universidade Estadual de Maringá, Maringá, Paraná 87020-900, Brazil.
[3] Christian Doppler Laboratory for Advanced Mid-Infrared Laser Spectroscopy in (Bio-)process Analytics, TU Wien, 1060 Wien, Austria.

*Correspondence and requests for materials should be addressed to georg.ramer@tuwien.ac.at.

Figure S1 illustrates how the temperature at the CaF_2/water interface responds to changes in excitation frequency and pulse duration. The time-dependent thermal behavior in Figure S1(a) clearly separates two components of the temperature field: a steady-state (DC) portion and an oscillating (AC) one. The steady-state increase reaches about 60 °C, showing that heat builds up continuously upon repeated laser excitation. The oscillatory part is much smaller, around 4 °C in amplitude, and represents the temperature fluctuations occurring at the modulation frequency. As the modulation frequency decreases, the amplitude of these oscillations grows. This happens because the system has more time to allow heat to spread and accumulate between pulses, leading to stronger thermal gradients and a more pronounced photothermal lens effect.

In Figure S1(b), the influence of pulse duration on the temperature change is shown. When the pulses become shorter, both the steady and oscillating temperature components diminish. Shorter pulses deposit less energy per cycle, resulting in lower overall heating and smaller temperature changes. With less energy delivered, both the average (DC) and periodic (AC) heating effects weaken, reducing the strength of the photothermal signal. Together, these results demonstrate that modulation frequency and pulse duration jointly control how heat accumulates and dissipates. Their interplay ultimately determines the size of the temperature gradients, and therefore the strength and behavior, of the O-PTIR photothermal response.

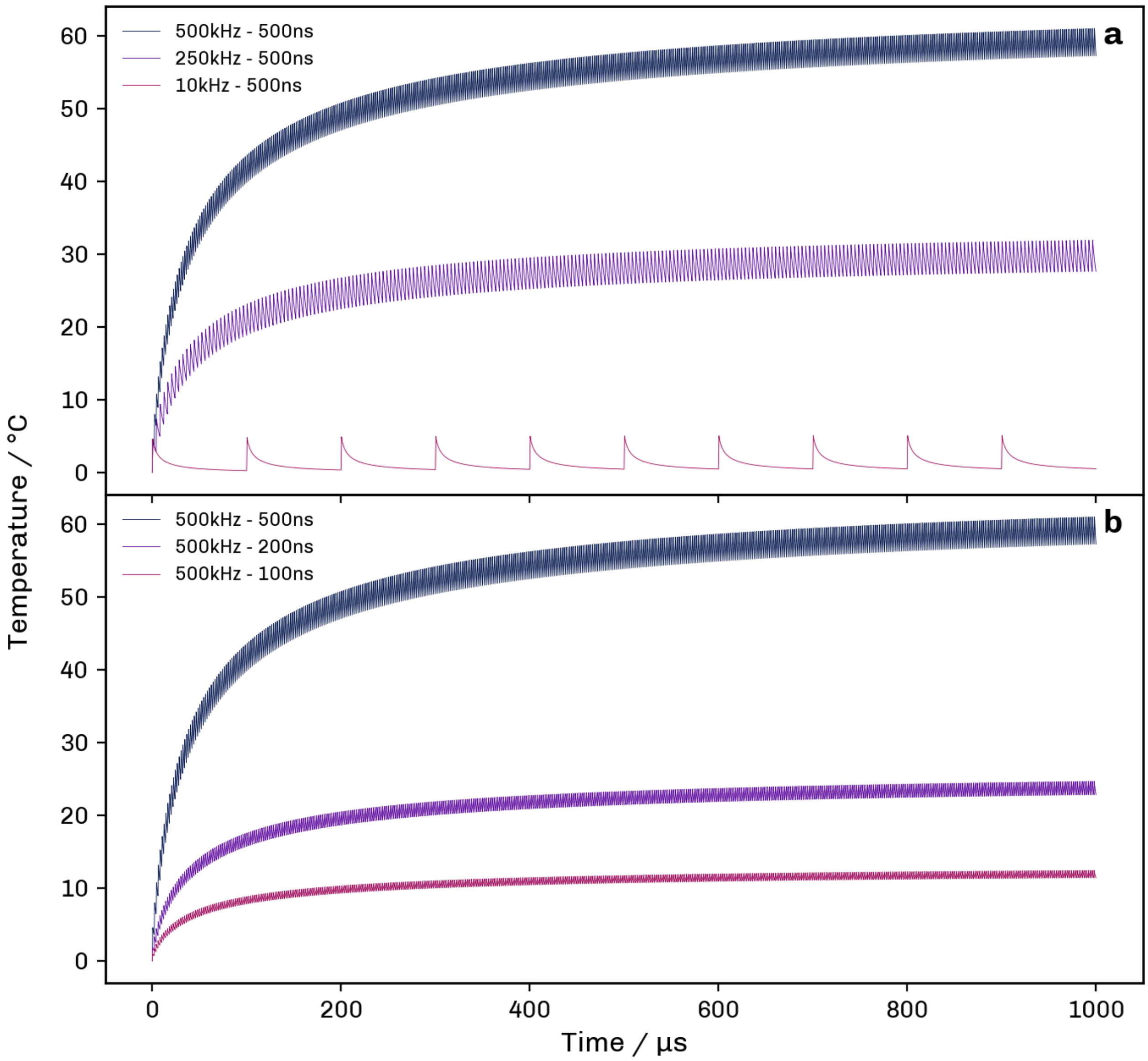

Figure S1: Temperature change at the CaF_2/water interface at the beam center ($r = 0$)for (a) different excitation frequencies and (b) different pulse durations calculated using Eq. 1.

Supporting Figure S2 shows that, at an excitation wavenumber of $1660\,\mathrm{cm}^{-1}$, the experimental and simulated transient dynamics vary only weakly with the axial position of the excitation focus. Because the excitation radiation is strongly attenuated within the first few micrometres of water, shifting the nominal focus primarily changes the excitation-beam radius and intensity distribution at the CaF_2/water interface, where most of the optical energy is absorbed.

Near the interface, heat diffuses through a heterogeneous environment comprising CaF_2 and water, which have different thermal diffusivities. Since thermal transport is faster in CaF_2 than in water, the resulting temperature and refractive-index fields extend into both materials. Changes in the nominal focal position modify the spatial distribution of the deposited energy and therefore the effective photothermal lens sampled by the probe beam. The slightly shorter transient times observed at larger focal offsets are qualitatively consistent with these changes in excitation geometry; however, the differences are small relative to the experimental variability and should not be interpreted as changes in the intrinsic thermal diffusivity of water.

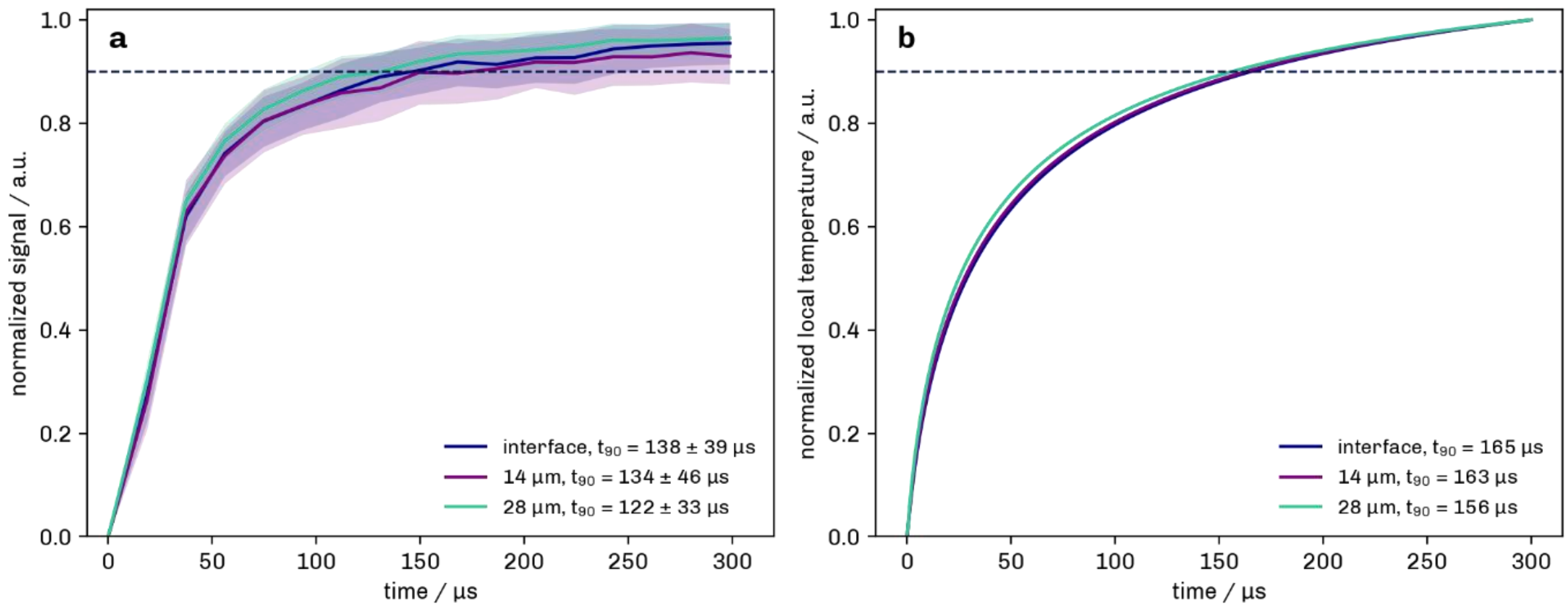

Figure S2: Experimental and simulated O-PTIR transients at the beam center ($r = 0$) at the CaF_2–water interface and at positions 14 µm and 28 µm inside the water phase. (a) measured transient O-PTIR signals. (b) corresponding simulated transients with 1660 cm^{-1} as excitation wavenumber. The similar temporal evolution across the interface and water positions indicates close agreement between experiment and simulation.
